# “We are actually being involved in management of the patient”: A qualitative exploration of experiences of students and faculty regarding the use of clinical simulation in Busitema University and Lira University

**DOI:** 10.21203/rs.3.rs-4242598/v1

**Published:** 2024-04-16

**Authors:** Joshua Epuitai, Pamella R. Adongo, Paul Oboth, Felister Apili, Edward Kumakech, Samuel Owusu-Sekyere, Julius N Wandabwa

**Affiliations:** Busitema University; Busitema University; Busitema University; Lira University; Lira University; Kwame Nkrumah University of Science & Technology Kumasi; Busitema University

**Keywords:** Clinical simulation, Perceptions, Undergraduate students, Teaching and Learning, Uganda

## Abstract

**Introduction:**

Experiences regarding the use of simulation in low-resource settings like Uganda where it has not taken root have not been explored. The purpose of the study was to explore the experiences of students, clinical staff, and faculty regarding the use of clinical simulation in teaching undergraduate students.

**Materials and methods:**

The study was conducted at Busitema and Lira Universities in Uganda. We conducted 20 in-depth interviews with the faculty staff and 10 focused group discussions with undergraduate Nursing, Midwifery, Medical and Anesthesia students. The study obtained ethical clearance from the Busitema University Research and Ethics Committee (BUFHS-2023–78) and Uganda National Council of Science and Technology (HS3027ES). Thematic analysis was used to analyze the data

**Results:**

Four themes emerged from the data. Simulation was seen to be about improvising and (return) demonstration. Concerns of realism were expressed including notions that simulation was not real, that simulation felt real and the extreme end that simulation tends to present the ideal setting. Perceived benefits of simulation include room for mistakes and immediate feedback, enhanced confidence and self-efficacy, enhanced acquisition of soft and clinical skills, prepares students for clinical placement, convenient and accessible. Concerns were expressed related to whether skills in clinical simulation would translate to clinical competence in the clinical setting

**Conclusion:**

Students perceived simulation to be beneficial. However, concerns about realism and transferability of skills to clinical settings were noted. Clarifying preconceived notions against the use of clinical simulation will enhance its utilization in educational settings where simulation is not readily embraced.

## Background

Clinical simulation is a critical pedagogical method of teaching clinical skills in which students are made to develop clinical competencies in a setting that mimics real-life clinical environment [1]. Simulation offers numerous superior benefits compared to the traditional model of teaching [2]. The advantage that clinical simulation offers is an opportunity for students to learn, re-learn, and make mistakes while uncompromising patient safety and reducing students’ performance anxiety [2–4]. Besides the acquisition of clinical psychomotor skills and compatibility with ethical principles, simulation enables students to acquire cognitive soft skills such as inter-professional collaboration, teamwork, and communication skills among many other skills which ordinarily were not possible in the traditional model of teaching [2, 5].

Although Simulation is widely used in developed countries, its utilization in resource limited settings such as Uganda is still low [4, 6]. In developing countries, simulation has often been limited to summative assessment during objective structured clinical examinations [6–8]. Concerns have been expressed against simulation especially that the skills acquired from simulation do not purportedly translate to clinical practice as well as the perceived effect that simulation reduces the number of times students will practice on real patients [3, 5, 8]. Subconsciously, the notion that simulation is a foreign western method of medical training that is not akin to the African belief system of medical pedagogy may have further limited its use in developing countries [9]. In Uganda, limited studies have generated context-based evidence vital to scaling up the utilization of clinical simulation especially in the context of interprofessional education and collaboration among health professionals. The study explored perceptions regarding the use of clinical simulation in training of undergraduate health professionals.

## Methods and Materials

### Study design

This was a qualitative study which used a grounded theory approach to understand stake-holders experiences and perceptions of simulation in our setting. We used qualitative methods to collect the data for the study.

### Study setting

The study was conducted in Busitema University Faculty of Health Sciences (BUFHS) and Lira University Faculty of Nursing and Midwifery. The two universities have skills laboratory and simulation center and curricular which provide students with opportunities to participate in clinical simulation sessions. BUFHS has undergraduate nursing, anesthesia, and medicine programs, while Lira University runs midwifery and medicine undergraduate programs.

### Study participants and sampling method

The study population for the side of the students comprised undergraduate students of nursing, anesthesia, midwifery and medicine programs. The students were in their clinical years of study from third to fifth year. The preceptors and faculty from BUFHS and Lira University were as well included in the study. Purposive sampling was used to select study participants. Purposive sampling was based on years of clinical experience, preceptorship and role of faculty staff at the university.

### Methods of data collection

We conducted 10 clinical simulation sessions with students in areas of maternal and child health, emergency and critical care. We used interviewer-guide for the focused group discussions (FGDs) and the in-depth interviews. The questions in the interviewer-guide included students’ and faculty members understanding of clinical simulation; experiences of students in a clinical simulation; students’ and faculty members’ attitudes, preferences, thoughts and perceptions towards simulation as a method of teaching; and perceptions of students and faculty members regarding whether clinical skills learned during a clinical simulation would translate to actual clinical competence when students are in a clinical setting. We conducted 10 focused group discussions (FGDs) with students to understand their perceptions regarding the use of clinical simulation as a teaching method. The FGDs comprised groups of 6–8 participants, totaling 65 students.

We conducted 20 in-depth interviews (IDIs) with the faculty members and preceptors in Lira University and Busitema University. The composition of faculty ranged from the level of teaching assistant to professorial level including the Deputy Dean, Heads of Department, and the Dean of the faculty. The research team (3 males, one female, nurses/midwives by profession) who participated in data collection were trained and had experience in conducting qualitative interview. Interviews were audio recorded after obtaining informed consent. The KIIs and IDIs lasted about 30 minutes while FGD took a duration of about 45 minutes to one hour.

### Data analysis and rigor of the study

The audio recordings were transcribed verbatim. A thematic data analysis approach, as described by Brauna and Clarke, was used to analyze the data [10]. This involved reading the transcripts for several times to become familiar with the data, generating initial codes, searching, reviewing and renaming of themes and presentation of the final report. A figure is provided for visual representation of interconnectedness of sub-themes and themes. The rigor of the study findings was guaranteed through triangulation of several methods of data collection (FGDs and IDI), while credibility was maintained by use of research personnel who were involved in simulation process.

### Ethical considerations

The study obtained ethical approval from Busitema University Research and Ethics Committee (BUFHS-2023–78) and Uganda National Council of Science and Technology (Reference number: HS3027ES). The study obtained written informed consent from the study participants.

## Results

### Theme 1: Simulation is demonstrating or improvising

Although the majority of the students and the faculty perceived simulation as the use of mannikins and models to mimic real life clinical scenarios (Table 1), some thought that simulation was about return demonstrations. Others thought that simulation involved improvising with models when real patients or clinical cases were not available on the ward.

“…they [faculty] have not understood what simulation is, …. even me before the training, I thought simulation is just demonstrating or part of improvising, not a clinical method of teaching. Because you don’t have a pregnant mother here or you don’t have a case scenario on ward, so you look for a model and demonstrate. That’s what they know about simulation as demonstrating or part of improvising”

(IDI-faculty).

### Theme 2: Concerns for realism

#### Sub-theme 1: Simulation does not feel real

Simulation was seen as not similar to the real clinical experience. This lack realism was seen to cause performance anxiety when students were on the real ward but also affect how students interacted with the simulated mannequins which negated transferability of skills from the simulation set up to the clinical environment.

“They normally say how it feels on the real patients is not how it feels on the model….they tend to have picked the skills [from simulation] but when on case scenarios with patients, some of them don’t do exact thing they demonstrated [when] on the ward”

(IDI-faculty).

“As you see, most of the times when you keep touching the model, you just feel it is a model and not a human being, you just touch the way you want, you become rough on it, you even tear…so when they come to patients, they start fearing patients because this one is now reality and you are working on somebody”

(IDI preceptor).

#### Sub-theme 2: Simulation feels real

Although the faculty and clinical preceptors perceived simulation as different from clinical environment, almost all students who participated in a simulation exercise felt that it mimicked a real-life clinical experience. Perceptions of realism of simulation among students were backed up with feelings of anxiety and other physical symptoms during the simulation exercise. Students noted that they felt they were really working on a patient and the whole simulation exercise felt like the routine activities that they were doing on the ward.

“If it is an emergency, you feel like you are really attending to an emergency, and sometimes the anxiety is actually there”

(FGD-student).

#### Sub-theme 3: Impractical to simulate everything

Although students admitted that simulation felt real, they thought that human interaction and experiences such as verbal and non-verbal cues could not be simulated. This was perceived to affect performance in the simulation and in the ward settings.

“Someone who is bleeding out a lot is not the same way we are going to simulate that scenario when talking to the caretaker. Here, you will just say calm down, things will be ok but there when you are looking at someone bleeding out. You can’t mention the word everything will be ok because you are not sure if things will end up well”

(FGD-student).

#### Sub-theme 4: Simulation presents the ideal

Concerns that simulation did not mimic the clinical setting was related to the perceived tendency for the simulation to present the ideal which did not reflect the clinical environment which often does not have the required equipment, medicines and supplies to be used.

“…..when you come to the simulation session I make sure that everything that you want is there but when you take them to the ward and present them to the same situation, I am very sure their performance will change because some things won’t be there, sometimes they even don’t know where to get them…..”

(IDI-faculty).

“Sometimes simulation is not up to simulation. Why? Simulation tends to present the ideal yet in actual sense our stations are never ideal. I might, for example, have simulation for cardiac arrested patient and the defibrillator is present…but in actual sense you find that you might do CPR for two hours without defibrillator around,…we should also look into having simulation whereby you are on a unit but somethings are not actually there. What do you do?”

(FGD-student).

### Theme 3: Perceived benefits of simulation

#### Sub-theme: Room for mistakes & feedback

Students and the faculty perceived simulation to provide room to learn from the mistakes but also identify weak students and areas of improvement. The use of models provided a safe environment for students to hone clinical skills without the risk of causing fatal medical errors while trying to learn.

“It is a low-risk environment, you are allowed to make mistakes and the results are not fatal: the patient does not suffer as a result of you trying to learn. And then the other thing is unlike what happens in real life, this can be slowed down, it can be broken down…”

(FGD-student).

“There is a debrief to know what you did right, what you missed…I remember I participated in a simulation doing pain management, I gave the patient morphine and I didn’t give a laxative so in the debrief, and up to now I am aware that a laxative should be given when you administer morphine”

(FGD-student).

Students valued the judgement free feedback given during simulation unlike in the ward where they would be emotionally and physically harassed for making a mistake.

“With this simulation as a student, if I made a mistake, I have a chance for correction and that is true because on the ward, should you make a mistake…you may be even beaten up…they shout at you and you even forget what you’re doing….”

(FGD-student).

“If you can see that something you can do can kill a patient in this case a dummy, you won’t be able to repeat that and if it was in the ward, you can imagine the guilt you can be feeling because of killing a patient”

(FGD-student).

#### Subtheme 2: Boosts self-efficacy and confidence

Simulation was seen to remove fear and anxiety when working on the real patients especially for students who were placed in the clinical environment for the first time. Simulation was perceived to give students a sense of confidence emanating from a feeling of having performed a similar procedure before during the simulation.

“For me I think the model gives students confidence. They develop that confidence before…When they are coming to the ward, that confidence is already in them, so they come when the fear is minimal. They have confidence that I can do this, I managed last time to pass NG tube on a dummy, I think I can also do this”

(FGD2 preceptor).

“…It has helped me develop the confidence in doing procedures before I go to the hospital”

(FGD-student).

#### Subtheme 3: Promotes acquisition of soft skills and clinical skills.

Students and faculty thought that simulation facilitated learning and acquisition of clinical skills because of its practical nature. As a result, simulation was thought to be better suited for teaching practical areas of clinical management. Simulation was thought to be more relevant for teaching emergency clinical skills where it was not feasible for bedside teaching as the focus was always to save the life of a patient.

“I think it helps to increase competence of students….”

(FGD-student).

“It enables the students learn how to manage emergencies because if you get an emergency on ward, you actually have no time to teach during that process….”

(IDI-faculty).

Besides clinical skills, simulation was perceived to promote acquisition of soft skills such as group learning, teamwork and communication.

“One shall learn how to work as a team. And then it also helps us to make quick thinking….it also tests the understanding…”

(FGD-students)

“The simulation brought out the image and that need for teamwork…”

(IDI-faculty).

#### Subtheme 4: Prepares students for clinical learning

Students and faculty alike perceived simulation as an entry point that prepares students to clinical work experience. The simulated environment provides students with confidence, knowledge and exposure to clinical ward.

“So, learning on the model is the first start. It gives them the first knowledge and the first skills before they start on a patient which is very important”

(FGD-preceptors).

“It has taken a stride in helping us learn a lot of things from here before going to wards”

(FGD-student).

In the University, students felt that transition from pre-clinical to clinical years was done in fast and abrupt way without adequate preparation for clinical exposure. As a result, the majority of students remarked that simulation should be introduced in the pre-clinical years to prepare students for clinical placement, while some thought that simulation is still relevant in clinical years.

“I would recommend it…for students who are transiting from pre-clinicals to clinicals…..and also it can help students who are already in clinicals to be like a daily reminder……”

(FGD-student).

#### Subtheme 5: Accessibility and convenience

Simulation was thought to be more practical for teaching clinical skills without violating ethical needs of patients, while simulation was attributed to be more efficient and provide hands on experience for teaching rare conditions in the ward.

“One is easy to use…if you have a group of 15 students and you want all of them to practise the skills, it would be hard to demonstrate the same skills on one patient”

(IDI-faculty).

“Today, I have learned to diagnose PPH and how to manage it in just a few minutes”

(FGD-student).

“It is very helpful since it is really a picture of bedside teaching and hands on. So, you realize that there are some conditions that you may not encounter in real life on ward but here and the experience was really nice”

(FGD-student).

### Theme 4: Concerns of whether skills in simulation translate to clinical competence in the clinical setting

#### Sub-theme 1: Skills in simulation do not translate clinical competence

When participants were asked whether the skills learned from simulation were transferable to the clinical setting, divergent views were expressed regarding the role of simulation in imbuing clinical competence to students. Some thought that simulation did not make students clinically competent because the models used in simulation were not real patients. Consequently, students were thought to be unable to interact and communicate with patients.

“…such students don’t [perform well], reason being that these models are not real patients”

(IDI preceptor).

#### Sub-theme 2: Simulation makes students clinically competent

Although perceived lack of realism was thought to undermine acquisition of clinical competencies, students perceived that the skills learned from simulation would translate to clinical competence. This was because simulation felt real to them which was underscored by experience of physical symptoms during simulation. Similar views were expressed by faculty who noted that they had observed transfer of skills from simulation to the clinical environment.

“It will translate into practice. It is a real-life experience. Today, one of our colleagues was sweating”

(FGD-student).

“Yes, I think so…. if they have done simulation sessions and have been able to practise that and put it into practise and that memory of the simulation will help deal with any incidence in theatre much more easily and I have seen that happen. So definitely the scenario helps, it translates from classroom-based teaching to clinical practise”

(IDI-faculty).

#### Sub-theme 3: Clinical simulation superior to other methods of teaching

When relating the role of simulation in transfer of clinical skills, students and faculty seemed to make comparison with the commonly used pedagogical methods of teaching and posed questions on whether there was transfer of clinical skills among traditional methods of teaching. Simulation was thought to be more superior than other pedagogical methods as it was more practical and involved direct hands-on experience. The faculty thought that simulation should not be taken as a sole method of clinical teaching but should be used in complementary with other teaching methods.

“….does a lecture translate to patient outcomes?….We tend to see it as if it’s a standalone, and that people are going to ask, if I do it, will I get better outcomes? I would like to think about it as not being a standalone, simulation is just one of the 101 delivery methods……..”

(IDI-faculty).

“Yes, I think because it is not like a lecture. Here, we are actually being involved in the management of a patient…..if you have done something before, there is a high probability you will be able to redo it even on a patient”

(FGD-student).

“…If we are having any scenario…when it comes to the part of managing, it is better to use simulation in trying to manage the patient so that the person can really understand what should be done”

(FGD-student).

## Discussion

The study explored the views of faculty, preceptors and students regarding the use of clinical simulation in teaching of clinical skills. Overall, simulation was not readily understood as a method of clinical teaching as it was perceived to mean demonstration or improvising. Although notions that simulation was not real were expressed among clinical preceptors and faculty, students who went through simulation noted that the simulation experience felt like a real clinical exposure as some of them were even anxious. Related to realism, clinical preceptors thought that the skills from simulation were not transferable to clinical environment which was contrary to perceptions of the faculty and the students. Faculty and students alike reported that simulation fosters acquisition of soft skills such as confidence, teamwork and self-efficacy as well as concrete clinical skills.

Clinical simulation has not been widely studied in sub-Saharan Africa[11]. Contrary to findings from previous studies [12–14], clinical simulation was conceptualized as demonstrations and improvising in our study. This maybe attributable to lack of exposure of clinical staff to simulation during their training as the students who had simulation experience and the faculty involved in use of simulation had a proper understanding of simulation. Conceptualization of simulation as demonstration highlights that simulation has not taken root in most of the training institutions in Uganda but also accounts for the traditional beliefs held against the use of clinical simulation. Participants thought that limiting simulation to demonstrations led to unintentional construction of simulation scenarios which did not mimic real-life clinical environment.

Realism in clinical simulation is critical in enabling students to fully participate in the simulated environment [15]. Consistent with previous studies [15, 16], simulation was regarded as a real-life clinical experience by students which was in stark contrast to clinical staff and faculty perceptions that simulation was far from reality. Although simulation is more than just models [16], the simulated models, who have no verbal and non-verbal expressions in them, were thought to be different with real patients which in itself was an inditement to the lack of high-fidelity models in the institution. Interestingly, simulation was perceived to present the ideal environment which was replete with all the equipment, and thus, was seen not to reflect the resource-constrained clinical environment where critical equipment, supplies and medicines may sometimes be lacking. The idealistic simulations were thought to negatively affect clinical performance of students when they were placed in a clinical setting as they would not be able to improvise and work with the available equipment. This underscores the need to balance the equipment available for simulation to reflect the limited resources in the clinical environment and the need to have equipment that ensure realism of clinical simulation experience in low- and middle-income countries.

Previous studies have underscored that students value clinical simulation for a number of reasons [16, 17]. Consistent with previous studies [15–17], both students and faculty alike thought that simulation provided a safe environment for practice which fostered room for mistakes, constructive feedback, confidence, self-efficacy, teamwork, group learning and ultimate acquisition of clinical skills. Faculty tend to give more attention to students during simulation [18], a finding which was related to calls in our study to use simulation in emergency situations where more priority is usually placed to saving the life of the patient with limited opportunities for learning after the management of the emergency. Unlike the hostile clinical environment and enormous consequences students would face in the clinical area for making a medical error, simulation was thought to promote learning from the mistakes and provide platform to repetitively master clinical skills in the setting of readily available judgement free feedback. The safe environment for practice boosted self-efficacy and confidence of students, a finding which is consistent with other studies [16, 18]. There were calls to introduce simulation in the pre-clinical years as the transition to clinical years were deemed to be swift without adequately preparing students with basic nursing skills. The need to introduce simulation in preclinical years was related to perception that simulation prepared students with prior knowledge, skills and attitude and particularly reduced anxiety and medical errors among students who were in the clinical area for their first time.

Currently, evidence is inconclusive regarding whether skills from simulation are transferable to clinical setting [11]. In our study, most of the students and faculty thought that the skills from simulation were transferable to clinical practice. Similar studies have equally reported perceived transferability of skills from simulation to clinical practice [19, 20]. In our study, perceptions that skills learned from simulation were transferable to clinical practice resulted from the practical aspect of simulation which was superior for teaching clinical skills than the currently used methods of teaching. A similar Opponents against the effectiveness of clinical simulation in making students clinically competent often perceived simulation as a standalone method instead of viewing it as complementary to other clinical methods of teaching. Despite use of high-fidelity equipment, students in Australia thought that clinical skills from simulation were not transferable to clinical practice because of dissimilar simulation-clinical environment, lack of opportunities to practice skills learned from simulation and difficulty attributing skills to simulation [20]. Transferability of skills from simulation to clinical practice, therefore, may not be clear-cut and may be determined by the setup of simulation, realism, students’ ability to reflect and the opportunity for students to practice [19].

## Conclusion

Simulation was mostly perceived as mimicking real life clinical environment. Some notions of simulation as focused on improvising and demonstrations were expressed. Some divergent views were expressed regarding realism as some thought simulation was not realistic while students and faculty involved in simulation perceived simulation as a real-life clinical experience. The perceived benefits of simulation included room for making mistakes, immediate feedback, enhancing confidence and self-efficacy, enhances acquisition of soft and clinical skills, prepares students for clinical placement, convenience and accessibility. Divergent notions were expressed regarding the role of simulation in enabling students become competent. The study findings have important implications for designing effective simulation scenarios but also for improving utilization of simulation in settings where simulation is not deeply entrenched.

## Figures and Tables

**Table 1:** Themes on experiences and perceptions regarding the use of clinical simulation

**Theme 1. Simulation seen as demonstration or improvising**	**Theme 2: Concerns of realism** **Lacks realism** **It feels real** **Impractical to simulate everything** **It feels more ideal**
**Theme 3. Perceived benefits of simulation** Room for making mistakes Provides room for constructive feedback Boosts confidence and self-efficacy Prepares students for clinical placement Available and readily accessible Saves time	**Theme 4. Concerns of whether skills in simulation translate to clinical competence in the clinical setting** It does not lead to clinical competence Simulation makes students competent Simulation is superior to other methods of teaching

## Data Availability

The datasets used and/or analyzed during the current study are available from the corresponding author on reasonable request.

## Abbreviations

BUFHs: Busitema University Faculty of Health Sciences
FGD: focused group discussions
IDI: in-depth interviews
REC: Research and Ethics Committee
